# A randomized controlled trial for a peer-facilitated telemedicine hepatitis c treatment intervention for people who use drugs in rural communities: study protocol for the “peer tele-HCV” study

**DOI:** 10.1186/s13722-023-00384-z

**Published:** 2023-05-27

**Authors:** Megan C. Herink, Andrew Seaman, Gillian Leichtling, Jessica E. Larsen, Tonhi Gailey, Ryan Cook, Ann Thomas, P. Todd Korthuis

**Affiliations:** 1 College of Pharmacy, Oregon State University / Oregon Health & Science University, Portland, USA; 2 Division of Addiction Medicine, Department of Internal Medicine, Oregon Health & Science University, Portland, USA; 3 Comagine Health, Portland, USA; 4 Oregon Health & Science University, Portland, USA; 5 Section of Addiction Medicine, Oregon Health & Science University, Portland, OR USA; 6 Oregon Health Authority, Salem, USA

**Keywords:** Hepatitis C virus, Peer-facilitated treatment, Direct acting antivirals, Telehealth, People who use drugs

## Abstract

**Background:**

Hepatitis C virus (HCV) transmission is primarily driven by injection drug use, and acute HCV infection rates are increased in rural communities with substantial barriers to care. Treatment of HCV in persons who use drugs (PWUD) is cost effective, decreases high risk behaviors and HCV transmission, and achieves high rates of treatment completion and sustained viral response. Adapting HCV care delivery to utilize peer support specialists, telemedicine technology, and streamlined testing and treatment strategies can better reach rural populations living with HCV.

**Methods:**

This is an open label, two-arm, non-blinded, randomized controlled trial designed to test the superiority of peer-facilitated and streamlined telemedicine HCV care (peer tele-HCV) compared to enhanced usual care (EUC) among PWUD in rural Oregon. In the intervention arm, peers conduct HCV screening in the community, facilitate pretreatment evaluation and linkage to telemedicine hepatitis C treatment providers, and support participants in HCV medication adherence. For participants assigned to EUC, peers facilitate pretreatment evaluation and referral to community-based treatment providers. The primary outcome is sustained virologic response at 12 weeks post treatment (SVR12). Secondary outcomes include: (1) HCV treatment initiation, (2) HCV treatment completion, (3) engagement with harm reduction resources, (4) rates of substance use, and (5) engagement in addiction treatment resources. The primary and secondary outcomes are analyzed using intention-to-treat (ITT) comparisons between telemedicine and EUC. A qualitative analysis will assess patient, peer, and clinician experiences of peer-facilitated telemedicine hepatitis C treatment.

**Discussion:**

This study uses a novel peer-based telemedicine delivery model with streamlined testing protocols to improve access to HCV treatment in rural communities with high rates of injection drug use and ongoing disease transmission. We hypothesize that the peer tele-HCV model will increase treatment initiation, treatment completion, SVR12 rates, and engagement with harm reduction services compared to EUC.

*Trial registration* This trial has been registered with ClinicalTrials.gov (clinicaltrials.gov NCT04798521)

**Supplementary Information:**

The online version contains supplementary material available at 10.1186/s13722-023-00384-z.

## Background

Hepatitis C virus (HCV) is a bloodborne virus that often results in chronic infection, leading to liver disease, including cirrhosis and hepatocellular carcinoma [1]. HCV transmission in the United States is primarily driven by injection drug use [2]. In 2018, 72% of new cases in the U.S. were associated with injection drug use and over 65% of cases occurred in persons aged 20–39 years [2]. The increase in opioid use disorder among young, nonurban people has fueled sharp rises in new HCV infections [3, 4]. Rural county characteristics associated with acute HCV infection rates include greater drug-overdose death rates, prescription opioid sales, proportion white non-Hispanic race/ethnicity, unemployment, lower per capita income, and limited buprenorphine prescribing capacity [4]. Oregon has the third highest chronic HCV prevalence in the United States and the second highest HCV-related mortality rate, with the highest prevalence concentrated in rural areas [5, 6]. COVID-19 pandemic-associated increases in drug use, overdoses, and potential decreased access to syringe services and substance use treatment may fuel subsequent rising infectious complications of injection drug use, including HCV [7–9].

Direct acting antivirals (DAAs) have revolutionized treatment of HCV, with cure or sustained virologic response at 12 weeks post treatment (SVR12) rates of ≥ 90%, shortened treatment duration, and fewer side effects than interferon-based treatments [10]. Current guidelines recommend treatment for all patients with chronic HCV infection, including for people who use drugs (PWUD) [10]. Treatment of HCV with DAAs in PWUD is cost effective, decreases high risk behaviors and HCV transmission, and achieves high rates of treatment completion and SVR12 [4, 11–13]. Still, PWUD face significant barriers to accessing treatment, such as restrictions for accessing DAAs, stigmatization, and limited access to venipuncture, noninvasive liver disease assessment and other preventive health services [12, 14–16]. This is especially true for people living in rural areas who often have inadequate HCV treatment access and long travel times to the nearest provider [17]. Additionally, those with active drug use have low awareness of treatment options and low HCV treatment uptake [18, 19].

New systems of care delivery that integrate HCV treatment in rural areas are urgently needed. Telehealth interventions, which directly connect patients to remote treatment providers, can support HCV treatment uptake in rural areas. One study found that telemedicine appointments with a HCV specialist integrated into an opioid treatment program for patients receiving methadone was effective, with 73% initiating DAAs and 93% of those initiating treatment achieving SVR12 [20]. Primary care providers have also successfully used telemedicine to expand the reach of HCV treatment among people engaged in primary care settings [21, 22]. However, few studies address the potential for telemedicine-assisted HCV treatment for PWUD who are not engaged in primary care or substance use disorder treatment, and none have implemented a peer-assisted approach.

Peer support specialists (“peers”) have lived experience in substance use and can reach, engage, and retain hard-to-reach populations [23]. Peer support specialists can directly engage PWUD, offer HCV screening, and link PWUD to treatment, though this approach has not yet been evaluated with a telehealth treatment model [24]. Streamlined testing and treatment strategies that limit laboratory and advanced fibrosis assessment reduce obstacles to delivering DAAs to PWUD [25]. This may increase the feasibility of peer-facilitated telemedicine-HCV treatment in this population.

The aim of this study is to test the efficacy of a peer-facilitated, streamlined telemedicine HCV treatment strategy (“peer tele-HCV”) versus facilitated referral to local HCV treatment (“enhanced usual care [EUC]”) for achieving and SVR12 among people who use drugs living with hepatitis C.

## Methods

We report the protocol (Version updated 6/17/2022) in concordance with the 2013 SPIRIT guidelines [26] (Additional file 1).

### Study design

This is an open label, two-arm, non-blinded, randomized controlled trial designed to test the superiority of streamlined peer tele-HCV compared to enhanced usual care (EUC) to achieve HCV cure (defined as SVR12) in PWUD in rural Oregon (clinicaltrials.gov NCT04798521). In-depth qualitative interviews are conducted with a sample of study participants to better assess their treatment experiences and attitudes. Peer and clinician focus groups are conducted to identify critical intervention components and lessons learned for external replication. The Oregon Health & Science University IRB (IRB00000471) reviewed and approved the study.

### Outcomes

The primary outcome is sustained virologic response as measured by an undetectable HCV RNA at 12 weeks post treatment. Secondary outcomes include: (1) HCV treatment initiation, (2) HCV treatment completion, (3) engagement with harm reduction resources, (4) rates of substance use, and (5) engagement in addiction treatment resources. Treatment completion is defined by a filled final DAA prescription and self-reported adherence of > 90% of DAA pills taken. Treatment initiation is defined as the first DAA prescription filled and self-report of taking the first pill. Participants who do not start treatment within 6 months are considered to have failed treatment (i.e., have a detectable HCV RNA level). Additional exploratory endpoints include treatment satisfaction (adapted from the single-item Medication Satisfaction Questionnaire [27]) and adherence to phone visits, defined as within 5 days before or after scheduled appointment.

Qualitative interviews are conducted by telephone with a sample of study participants to assess participants’ experiences with the telemedicine-HCV treatment intervention, including satisfaction with care, barriers to treatment initiation and adherence, and the role of peers in facilitating laboratory and telemedicine appointments, medication access, and adherence. A virtual focus group with peers assesses tele-HCV treatment barriers and facilitators, peer specialist activities, and advantages and disadvantages of tele-HCV. A virtual focus group with tele-HCV clinicians assesses peer-facilitated tele-HCV procedures; experiences engaging with specialty pharmacies, payers, peers, and patients; and clinical considerations.

### Setting and participants

This study takes place in rural communities in Oregon. The high prevalence of HCV in Oregon, significant percentage of the total population living in rural areas, and lack of specialist services outside of the Portland metro area make it an attractive location for the intervention [6]. All of the study’s peer specialists are certified by the state of Oregon, which includes completion of a week-long state-approved training program and biannual re-training and recertification. The study’s peers are also invited to participate in a monthly learning collaborative for peers statewide doing similar work (e.g. harm reduction and recovery support and linkage to hepatitis C testing and treatment) and an annual peer conference. Study peers participate in weekly full team meetings to talk through participant status and next steps, and the investigators conduct regular site visits to review documentation and observe and discuss procedures.

Peer support specialists and research assistants recruit PWUD who live in rural Oregon counties with high rates of overdose and hepatitis C infection to participate in this study. Rural counties with local syringe service programs (SSPs) providing both peer support specialist services and HIV/HCV screening are included as study sites: Douglas, Lane, Josephine, Coos, Curry, and Umatilla counties. Community-based SSPs serve as initial recruitment sites and are supplemented with community settings (e.g., parks, homeless shelters, community events). Participants are eligible for inclusion if they: live in the study area, have injected drugs, or used recreational opioids without injection in the last 90 days, are 18 years of age or older, have a positive HCV RNA, are enrolled in health insurance, and are interested in treatment. Participants are excluded if they have decompensated cirrhosis, defined as Child–Turcotte–Pugh (CTP [28]) score of 7 or greater (CTP B or C cirrhosis), or are pregnant or breastfeeding.

Participants are recruited through local SSPs, direct community outreach (e.g., parks, homeless shelters), and participant referral (respondent driven sampling). Research staff and peer coordinators encourage participants to use their personal networks to invite individuals for initial screening and participation. Participants receive cash incentives totaling $215 for survey completion and other study activities, including baseline and follow up surveys, blood draws, and treatment initiation visits.

### Procedures and follow-up

**Fig. 1:**
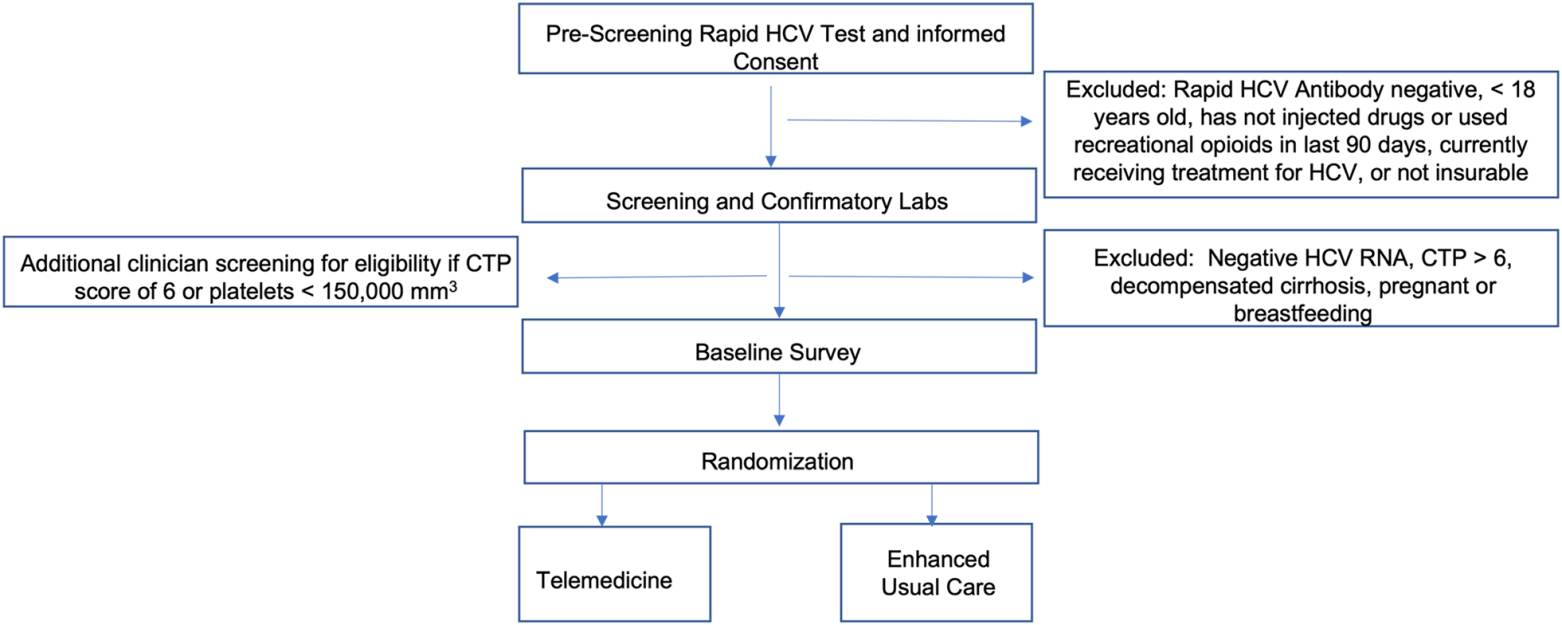
Screening and randomization

**Fig. 2:**
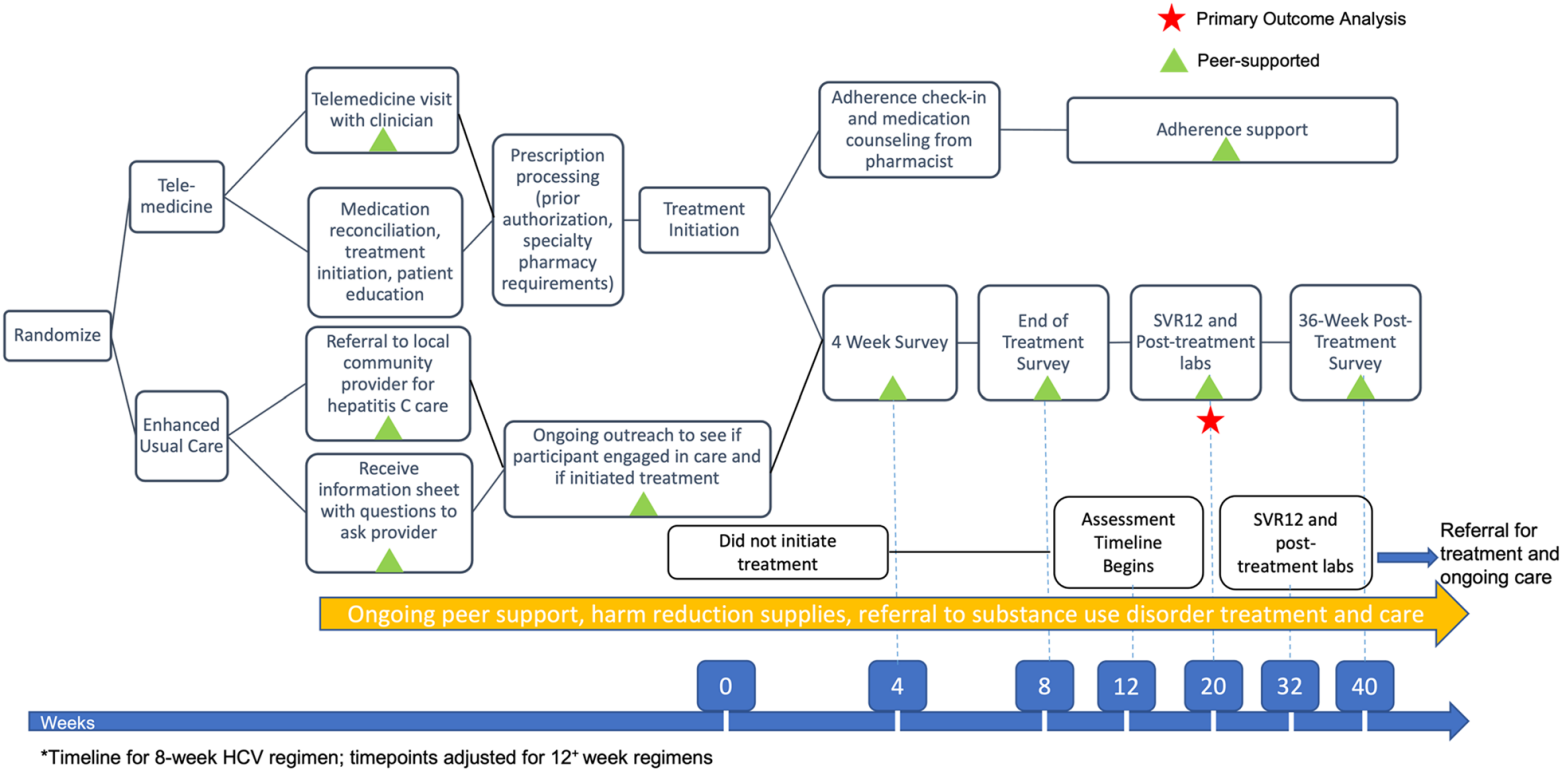
Trial design

Figures 1 and 2 depict participant procedures and study timeline. Pre-screening for eligibility includes a rapid HCV antibody test by local study staff or self-report from the participant of current HCV in addition to meeting other inclusion criteria. An Information Sheet Consent Form establishes consent for HCV rapid antibody testing during pre-screening. The OraSure® HCV Rapid Antibody test is a finger prick that can either be administered by study staff or self-administered, depending on participant or staff preference. Prior to the rapid HCV test, participants complete a full written consent form (see Additional file 2).

If the pre-screening HCV rapid test or self-report of chronic active hepatitis C is positive and they meet other inclusion criteria, peers complete the “Lab Form Determination” form to determine which standing order form is required (see Additional file 2: Table S1). Peers then accompany them to a local lab for confirmatory HCV RNA and additional baseline pretreatment evaluation by standing order (Table 1) to confirm eligibility. Peer support specialists can provide transportation and assist the participant in enrolling in health insurance at this time if necessary. The evaluation protocol streamlines lab testing and minimizes imaging assessments for cirrhosis to decrease barriers to treatment initiation and evaluation costs, in keeping with calls for simplified treatment evaluations [25]. Study investigators created standing lab orders for treatment naïve, treatment experienced, and women under 50, that allow for a single blood draw to assess appropriateness for DAA treatment. HCV genotypes are not routinely performed, unless the participant has a history of past DAA treatment failure. Women under the age of 50 are also screened for pregnancy. Rather than conduct imaging to assess for cirrhosis, participants with platelet counts of less than 150,000 per mcL or laboratory-based CTP score of 6 undergo a study clinician evaluation (see Additional file 2: Table S2) over the telephone exploring the likelihood of decompensated cirrhosis to determine eligibility for randomization. Participants who, in the opinion of the study clinician, have symptoms of decompensated cirrhosis, are referred for local ultrasound. All other participants are confirmed to be eligible for randomization without additional testing. Once eligibility is confirmed, all participants complete a baseline survey (Table 2) that includes questions regarding medical care history, barriers to treatment, engagement with harm reduction and substance use treatment, current substance use, perceived stigma of drug use, and HCV treatment history. Surveys assess use of non-prescribed prescription opioids, illicit opioids including fentanyl, methamphetamines, and other illicit substances, as well as the route of administration. Similar survey questions are asked at week 4 of treatment, end of treatment, at 12 weeks post treatment, and at 36 weeks post treatment. Follow-up surveys also ask about medication initiation, adherence, and changes in substance use and addiction treatment.

**Table 1:** Laboratory and other participant assessments collected

Assessment	Pre-screen	Screen	Baseline (week 0)	Week 4	End of treatment	12-week post-treatment	As needed
Rapid HCV test	X						
CTP assessment		X					
Participant survey	X		X	X	X	X	
HCV RNA		X				X	
Hepatitis B surface antigen		X					
Hepatitis B surface antibody		X					
Hepatitis B core antibody		X					
Hepatitis A antibody		X					
HIV antigen/antibody		X					
Complete metabolic panel		X				X	
Complete blood count		X				X	
INR/prothrombin		X					
B-HCG (women < 50 only)		X					
Ultrasound							X
HCV genotype (treatment experienced) and resistance testing							X
Medication counseling			X	X			

*B-HCG* beta-human chorionic gonadotropin, *CTP* Child–Turcotte–Pugh, *HCV* hepatitis C virus, *HIV* human immunodeficiency virus, *INR* international normalized ratio

**Table 2:** Baseline surveys and sample questions

Demographics
• What is your gender and race?
• How much school have you finished?
• What are your main sources of income over the last 6 months?
• Have you been homeless in the past 6 months?
• Do you currently have health insurance?
Baseline HCV questions
• Have you ever been tested for HCV?
• Have you ever been told you had HCV?
• In the last 30 days, have you seen a medical provider for your hepatitis C infection?
Access to health
• Do you have a way to get to medical appointments?
• Have you ever attempted to receive medications for HCV?
• What is your understanding of why you were able to access treatment for HCV in the past?
• What is the main place you have received medical care in the past 6 months?
Substance use
• Currently, which is your drug of choice for getting high?
• How many of the last 30 days have you injected drugs or used other illicit drugs?
• Have you ever overdosed?
Injection behavior
• On average in the last 30 days, how often have you injected any drug?
• Where have you gotten syringes or needles in the last 30 days?
• In the past 30 days, how many times did you get new syringes or needles, cottons, or cookers from a syringe or needle exchange program?
• How many times in the past 30 days did you use a syringe or needle that you know was used by somebody else?
• How many times on average do you reuse a syringe?
Addiction treatment
• In the last 30 days, have you gotten any treatment or help for an addiction problem?
• How do you feel about your drug use (e.g. ashamed, fear of losing friends or family?

Study data are collected and managed using REDCap electronic data capture tools hosted at OHSU [29, 30]. REDCap (Research Electronic Data Capture) is a secure, web-based software platform designed to support data capture for research studies, providing (1) an intuitive interface for validated data capture; (2) audit trails for tracking data manipulation and export procedures; (3) automated export procedures for seamless data downloads to common statistical packages; and (4) procedures for data integration and interoperability with external sources. Following completion of consent and baseline assessments, participants are randomized by study staff and allocated to a study arm using a centralized random number assignment assigned through REDCap. Prescreen, baseline and follow-up surveys collected in the field or via phone are first documented on paper forms. These are then entered into REDCap by the research assistant. The source documents are kept in a locked file cabinet in a secure location. Access to de-identified data in REDcap are limited to IRB-approved study staff that interact with participants. Participants who indicate willingness to be contacted for future studies in the consent form may be contacted and offered participation in future studies. Records are routinely audited by the investigator, with the support of the National drug abuse Treatment Clinical Trials Network (CTN) Western States Node data monitoring resources.

Throughout the study, peers offer harm reduction supplies (sterile syringes and works, fentanyl test strips, and naloxone overdose rescue kits) and substance use disorder treatment linkage to participants in both study arms.

### Intervention: tele-HCV arm

Participants randomized to the peer tele-HCV treatment intervention arm are scheduled for telemedicine visit and HCV assessment by a study clinician (physician, nurse practitioner, or clinical pharmacist) as soon as possible following randomization, with the goal of same-day appointments. For most participants, this is also the treatment initiation visit. The peer facilitates telemedicine visits using the chosen web platform of the participant. Telemedicine visits are conducted in a private location where the participant is comfortable. This could include the SSP office where the peers work, but can also occur at any location with adequate cellular-based internet (e.g., a park, car, or the participant’s home). Peer support specialists carry a tablet with internet connectivity to access the telemedicine visit if the participant does not have their own access. If additional tests are necessary for clinical decision making, peers assist participants in navigating health system barriers and arrange a second appointment with the telemedicine HCV treatment provider.

The telemedicine study clinician performs a standard of care hepatitis C treatment initiation history and evaluation and submits a prescription for an appropriate pangenotypic DAA treatment regimen (glecaprevir + pibrentasvir or sofosbuvir + velpatasvir per Oregon Health Authority prior authorization criteria) based on comorbidities, side effects, patient preference and insurance coverage. The study pharmacist assesses barriers to adherence, reviews medications for drug-drug interactions, reviews side effects, and provides patient education. Prior authorizations are completed and submitted by the study pharmacist.

All participants initiating treatment are prescribed DAA treatment for 4 weeks at a time. Most participants require two separate dispensations at week 0 and week 4 for a total of 8 weeks of medications, with a small subset requiring a third, 4-week dispensation for a total of 12 weeks of therapy. Medications are mailed to a home address or to the SSP office to be stored in a secure locker until the participant picks up the medication. The study clinical pharmacist contacts the participant by telephone or telemedicine visit at week 0 and week 4 to (1) determine medication tolerance, (2) assess adherence and (3) dispense medications by mail or ensure medications received, depending on the participant’s insurance. If the pharmacist is unable to connect with the participant after 3 attempts, treatment will not be delayed, and the peer will continue to assist in receiving all refills. Peers assist participants in keeping telemedicine appointments and in navigating medication pick up or storage, if not mailed directly to the home. If problems or side effects arise during treatment, participants call the research assistant or peers, who connects them to a study clinician. The study clinician will evaluate for considerations for discontinuation of therapy (e.g., evidence of new decompensated cirrhosis) and advise participants on management of treatment interruptions consistent with current guidelines [31]. If there is a significant gap in therapy (≥ 21 days), SVR12 from last day on treatment is collected and included in primary care analysis. SVR12 rates for participants in both arms with partial treatment completion will also be reported separately in the results.

Peers facilitate repeat HCV lab testing at 12 weeks following the end of treatment and relay results to the participant in the SVR12 follow-up visit, along with completion of follow-up surveys. End of treatment date is defined as the day the participant last took a DAA medication, as determined in the 8 week follow up survey or earlier communication with research staff. Those successfully achieving SVR12 are counseled on ongoing harm reduction methods to avoid reinfection. Those with persistent HCV viremia at 12 weeks post-treatment are referred to telemedicine treatment providers for treatment re-initiation outside of the study. All participants are scheduled for a 36-week post end of treatment survey visit.

### Control: enhanced usual care

Following completion of full pretreatment evaluation and study inclusion, research staff refer participants randomized to the EUC arm to a local community health clinic with experience treating hepatitis C to engage in treatment. EUC patients are encouraged to engage with local primary care and health plan resources and receive an information sheet on optional clinics to attend. The information sheet includes a list of questions participants are encouraged to take with them to their visit. Research assistants outreach to participants regularly to determine if they have engaged in local health resources, schedule assessment visits, and request a bidirectional release of information. Research staff call the clinic or participant periodically to ask if the participant has initiated treatment. After 3 months of no treatment initiation, the participant begins survey and other outcome data collection as if the participant had started treatment at that time. Peers facilitate SVR12 laboratory testing at 12 weeks after actual or intended end of treatment by study convention, depending on whether treatment is initiated. Local HCV referral providers are encouraged to participate in the OHSU Hepatitis C Elimination Extension for Community Healthcare Outcomes (ECHO) program but are not study participants. The hepatitis C ECHO is an interactive education and capacity building model expanding HCV treatment expertise to rural communities [32].

In either arm, participants who do not initiate treatment by 12 weeks after randomization begin their assessment timeline, mirroring the treatment timeline for an 8-week direct-acting antiviral regimen. For these participants, the primary study outcome SVR12 is assessed at 32 weeks after randomization, corresponding with 12 weeks after completion of 8 weeks of treatment. If participants initiate therapy between 12 and 24 weeks post randomization, the follow up and SVR12 dates are regenerated. Participants who do not start treatment within 6 months of randomization will be considered treatment failures.

## Sample size and power analysis

Based on pilot data showing 19% of known positives in the study region have been treated and an estimated spontaneous clearance rate of 25% over 36 weeks of follow up [33], we assume a HCV SVR-12 rate of 44% in those assigned to EUC. We first set the total sample size at 200 based on feasibility of recruitment, study protocol, and budget. We then determined that we would have 80% power to detect a 19% increase in SVR-12 with the intervention (i.e., an SVR-12 rate of 63% in the intervention group). This minimally detectable effect size and intervention arm SVR-12 rate was deemed clinically relevant, plausible with our intervention, and consistent with other accessible HCV care interventions among PWUD [34, 35]. Power calculations assume a two-sided hypothesis test at alpha = 0.05.

## Qualitative evaluation

One-on-one qualitative interviews are conducted by telephone with a sample of up to 35 purposively selected study participants to gain a better understanding of the perceptions and experiences of telemedicine hepatitis C treatment. Participants are stratified by treatment site (county) and medication adherence status and randomly selected within strata. An iterative process is used to assess variation and saturation, and additional information-rich cases are included in the sample of needed. Interviews are conducted by trained qualitative researchers and assess patient satisfaction and perceived quality of care; barriers and facilitators to medication initiation and adherence; the role of peers in facilitating laboratory and telemedicine appointments, medication access, and adherence; and how telemedicine impacted engagement with other services.

We also conduct virtual focus groups with peers and clinicians with a semi-structured interview guide to identify critical intervention components and lessons learned for external replication. The peer support specialist focus group assesses tele-HCV and HCV treatment barriers and facilitators, peer specialist activities and adaptations, and advantages and disadvantages of tele-HCV. The clinician focus group assesses peer-assisted tele-HCV procedures; barriers and facilitators; experiences engaging with specialty pharmacies, payers, and peers; and clinical considerations related to tele-HCV.

## Statistical analysis

### Primary and secondary outcomes

The primary and secondary outcomes are analyzed using intention-to-treat (ITT) comparisons between telemedicine and EUC. Because of the relatively small sample size, we compare baseline covariate distributions (demographics, etc.) between randomized groups using standardized mean differences (SMDs) and bivariate statistical tests (e.g., t-tests, Chi-square tests). If a significant imbalance in any variable is noted (i.e., a SMD > 0.25 and p < 0.2), we will include that variable as a covariate in future analyses.

**Fig. 3:**
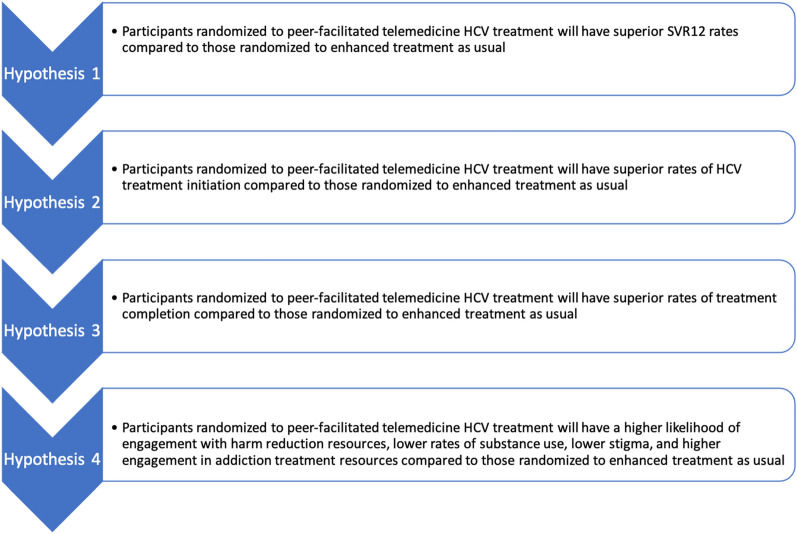
Study hypotheses

The primary analysis is a logistic regression model of SVR-12 (binary) as the outcome and randomized group as the covariate of interest. Additional baseline covariates are included per the criteria outlined above. Missing data are considered non-suppressed (i.e., SVR-12 not met). Secondary analyses utilize generalized linear (mixed) models (GLMMs) with distributions and link functions appropriate for the distribution of the outcome variable. If more than one timepoint is included in the analysis, models will include person-level random intercepts to account for repeated measurements on subjects. Hypotheses (Fig. 3) are tested using between-group comparisons at each timepoint of interest, or within-group comparisons across timepoints. Sensitivity analyses includes a per-protocol analysis and a repeat of the main analysis using multiple imputation of missing data.

### Qualitative analysis

Audio-recorded interviews and focus groups are professionally transcribed by a professional contracted transcriptionist and uploaded into qualitative analysis software (NVivo™). Using the integrated framework of access to HCV care, the analysis approach includes a coding structure and an inductive thematic process to identify emergent themes [36, 37]. Research staff create a coding scheme, conduct coding, and revise in an iterative process that has been successfully implemented by the study team in other mixed-methods projects. The qualitative data provides complementary information to illuminate and expand quantitative findings, including survey results and measures of treatment initiation and completion.

## Discussion

This study evaluates the efficacy of a novel streamlined delivery model to expand access to hepatitis C treatment for PWUD in rural communities. Our intervention includes multiple innovative components that remove barriers for treatment and create a pathway to treatment for this population. First, a peer-facilitated treatment approach recruits, engages, and creates a sustainable treatment approach for PWUD in rural counties. Peer supported treatment models can improve engagement of patients with chronic HCV, including PWUD, and reduce unsafe injection behaviors [38, 39]. In our study, peers recruit non-treatment-seeking PWUD, who typically have little contact with the healthcare providers, and help them navigate the complex healthcare system. Peers can assist with transportation to local lab facilities, facilitate telemedicine appointments, support DAA receipt and adherence, provide harm reduction resources and supplies, resolve insurance and payment issues, and engage in treatment options for substance use, if desired. We predict that allowing peers to use a wide range of communication methods with participants (e.g. texting, Facebook Messenger, street outreach) will address known telecommunication barriers and increase the likelihood of treatment initiation.

Second, offering telemedicine visits to participants and streamlining screening and monitoring removes unnecessary barriers to care and limits the number of steps required between diagnosis and treatment initiation. While treatment of HCV with the DAAs has expanded from specialty to primary care settings, rural community provider hesitancy to treat PWUD due to concerns for adherence, follow-up, and re-infection remains a barrier [19]. Our streamlined pre-treatment evaluation uses a standard lab form that are pre-signed by providers and minimizes more invasive testing, including liver imaging. Our approach utilizes non-invasive fibrosis surrogates, including Fibrosis-4 (FIB-4) or Aspartate aminotransferase to Platelet Ratio Index, and includes a standardized approach for clinicians to evaluate presence of decompensated cirrhosis in patients with elevated biomarkers associated with more advanced disease. Our lab form determination work sheet (Additional file 1) empowers peers to determine what lab form is needed for appropriate treatment pre-evaluation. Previous studies have shown decreased time to treatment initiation, cost effectiveness and an expedited care cascade progression with streamlined and non-invasive approaches [25, 40].

Telemedicine offers a low-cost intervention for reaching PWUD in rural communities. Telehealth interventions have been shown to be effective for PWUD with HCV [20, 41]. However, most studies integrate HCV care into an opioid use disorder treatment program, which limits generalizability to those actively engaged in treatment for opioid use disorder. Peers bridge access to telemedicine hepatitis C services for PWUD outside of the healthcare system and support PWUD throughout treatment. Our study adds to previous approaches by using peers to directly engage people who are actively injecting drugs—an essential population for achieving HCV elimination [18]. Additionally, we expect a significant portion of participants to have unstable housing, which has been associated with lower uptake of HCV testing and treatment [42]. Third, our interdisciplinary telemedicine treatment model broadens the types of health professionals providing care in the primary care setting to improve access and decrease demand on specialist providers [21, 43, 44]. To meet hepatitis C elimination goals, expansions of the treatment team are needed. Additional data supports the safety and efficacy of HCV delivery models including non-physician providers and clinical pharmacists [45–48]. Our approach utilizes clinical pharmacists through collaborative practice agreements and expands the capacity to treat individuals living with HCV in hard-to-reach communities with the support of the Oregon ECHO Network.

Our proposed trial may include several potential limitations. First, we are not able to blind participants or investigators to treatment assignment in this test of two treatment delivery strategies. The effectiveness of DAA’s, however, are well-established. Participants who fail to engage in EUC will be offered alternative treatment pathways. Second, our study population is likely to reflect the majority-white race/ethnicity demographics of Oregon. To address this, our community-based peer organizations will prioritize minority race/ethnicity peers to enhance connections to non-white populations. Third, we carefully considered the impact of a usual condition, where PWUD are unlikely to engage in treatment through referral community providers. We consequently compare the intervention to “enhanced usual care”, in which all participants benefit from peer SSPs and assistance with HCV screening and support completion of laboratory pretreatment evaluation—a major hurdle for engaging in community-based HCV treatment. Finally, high rates of houselessness, transportation and telecommunication barriers, and community stigma among PWUD in rural Oregon create challenges for continued engagement that may impact our ability to collect primary and secondary endpoints.

## Conclusion

This study will test the superiority of a novel peer supported telehealth HCV delivery model compared to enhanced usual care on HCV treatment initiation and cure in PWUD in rural Oregon. These proposed interventions are hypothesized to increase SVR12 rates and engagement with harm reduction services. Results of this study will be disseminated to help other organizations create more sustainable and streamlined pathways to treat and cure those with limited access to HCV treatments.

## Supplementary Information

**Additional file 1:** Clinical Trial Protocol SPIRIT Checklist.

**Additional file 2:** Supplementary Material.

## Data Availability

Not applicable (study protocol only).

## Abbreviations

CTN: Clinical Trials Network
CTP: Child–Turcotte–Pugh
DAA: Direct acting antivirals
ECHO: Extension for Community Healthcare Outcomes
EUC: Enhanced usual care
FIB-4: Fibrosis-4
HCV: Hepatitis C virus
ITT: Intention to treat
Peers: Peer support specialists
PWUD: People who use drugs
SVR12: Sustained virologic response at 12 weeks post treatment
SSP: Syringe service programs
REDCap: Research electronic data capture
SMD: Standardized mean differences
Tele-HCV: Telemedicine care

## References

[1] Rosen HR (2011). Clinical practice. Chronic hepatitis C infection. N Engl J Med.

[2] Centers for Disease Control and Prevention. Viral hepatitis surveillance—United States, 2018. https://www.cdc.gov/hepatitis/statistics/SurveillanceRpts.htm. Accessed 22 Apr 2021.

[3] Perlman DC, Jordan AE (2018). The syndemic of opioid misuse, overdose, HCV, and HIV: structural-level causes and interventions. Curr HIV/AIDS Rep.

[4] Van Handel MM, Rose CE, Hallisey EJ (2016). County-level vulnerability assessment for rapid dissemination of HIV or HCV infections among persons who inject drugs, United States. J Acquir Immune Defic Syndr.

[5] Oregon Health Authority Communicable Disease Control. Chronic Heaptitis C. 2019. https://www.oregon.gov/OHA/PH/ABOUT/Documents/indicators/hepatitisc.pdf. Accessed 20 Apr 2021.

[6] Oregon Health Authority Public Health Division. Hepatitis C infections in Oregon. 2017. https://www.oregon.gov/oha/PH/DISEASESCONDITIONS/HIVSTDVIRALHEPATITIS/ADULTVIRALHEPATITIS/Documents/Hepatitis-C-in-Oregon.pdf. Accessed 29 June 2022.

[7] Glick SN, Prohaska SM, LaKosky PA, Juarez AM, Corcorran MA, Des Jarlais DC (2020). The impact of COVID-19 on syringe services programs in the United States. AIDS Behav.

[8] Seaman A, Leichtling G, Stack E (2021). Harm reduction and adaptations among PWUD in rural Oregon during COVID-19. AIDS Behav.

[9] Blach S, Kondili LA, Aghemo A (2021). Impact of COVID-19 on global HCV elimination efforts. J Hepatol.

[10] AASLD-IDSA HCV Guidance Panel (2018). Hepatitis C guidance 2018 update: AASLD-IDSA recommendations for testing, managing, and treating hepatitis C virus infection. Clin Infect Dis.

[11] Williams BE, Nelons D, Seaman A (2019). Life projects: the transformative potential of direct-acting antiviral treatment for hepatitis C among people who inject drugs. Int J Drug Policy.

[12] Barbosa C, Fraser H, Hoerger TJ (2019). Cost-effectiveness of scaling-up HCV prevention and treatment in the United States for people who inject drugs. Addiction.

[13] Hajarizadeh B, Cunningham EB, Reid H, Law M, Dore GJ, Grebely J (2018). Direct-acting antiviral treatment for hepatitis C among people who use or inject drugs: a systematic review and meta-analysis. Lancet Gastroenterol Hepatol.

[14] Arain A, Robaeys G (2014). Eligibility of persons who inject drugs for treatment of hepatitis C virus infection. World J Gastroenterol.

[15] Day E, Hellard M, Treloar C (2019). Hepatitis C elimination among people who inject drugs: challenges and recommendations for action within a health systems framework. Liver Int.

[16] Litwin AH, Drolet M, Nwankwo C (2019). Perceived barriers related to testing, management and treatment of HCV infection among physicians prescribing opioid agonist therapy: the C-SCOPE study. J Viral Hepatitis.

[17] Havens JR, Walsh SL, Korthuis PT, Fiellin DA (2018). Implementing treatment of opioid-use disorder in rural settings: a focus on HIV and hepatitis C prevention and treatment. Curr HIV/AIDS Rep.

[18] Falade-Nwulia O, Gicquelais RE, Astemborski J (2020). Hepatitis C treatment uptake among people who inject drugs in the oral direct-acting antiviral era. Liver Int.

[19] Falade-Nwulia O, Irvin R, Merkow A (2019). Barriers and facilitators of hepatitis C treatment uptake among people who inject drugs enrolled in opioid treatment programs in Baltimore. J Subst Abuse Treat.

[20] Talal AH, Andrews P, McLeod A (2019). Integrated, co-located, telemedicine-based treatment approaches for hepatitis C virus management in opioid use disorder patients on methadone. Clin Infect Dis.

[21] Arora S, Thornton K, Murata G (2011). Outcomes of treatment for hepatitis C virus infection by primary care providers. N Engl J Med.

[22] Beste LA, Glorioso TJ, Ho PM (2017). Telemedicine specialty support promotes hepatitis C treatment by primary care providers in the department of veterans affairs. Am J Med.

[23] Stack E, Hildebran C, Leichtling G (2022). Peer recovery support services across the continuum: in community, hospital, corrections, and treatment and recovery agency settings—a narrative review. J Addict Med.

[24] Bruggmann P, Litwin AH (2013). Models of care for the management of hepatitis C virus among people who inject drugs: one size does not fit all. Clin Infect Dis.

[25] Seaman A, King CA, Kaser T (2021). A hepatitis C elimination model in healthcare for the homeless organization: a novel reflexive laboratory algorithm and equity assessment. Int J Drug Policy.

[26] Chan AW, Tetzlaff JM, Gøtzsche PC (2013). SPIRIT 2013 explanation and elaboration: guidance for protocols of clinical trials. BMJ.

[27] Kalali A (1999). Patient satisfaction with, and acceptability of, atypical antipsychotics. Curr Med Res Opin.

[28] Pugh RN, Murray-Lyon IM, Dawson JL, Pietroni MC, Williams R (1973). Transection of the oesophagus for bleeding oesophageal varices. Br J Surg.

[29] Harris PA, Taylor R, Minor BL (2019). The REDCap consortium: building an international community of software platform partners. J Biomed Inform.

[30] Harris PA, Taylor R, Thielke R, Payne J, Gonzalez N, Conde JG (2009). Research electronic data capture (REDCap)—a metadata-driven methodology and workflow process for providing translational research informatics support. J Biomed Inform.

[31] AASLD-IDSA. Recommendations for testing, managing, and treating hepatitis C. http://www.hcvguidelines.org. Accessed 20 Nov 2021.

[32] Arora S, Kalishman S, Thornton K (2010). Expanding access to hepatitis C virus treatment—extension for community healthcare outcomes (ECHO) project: disruptive innovation in specialty care. Hepatology.

[33] Bulteel N, Partha Sarathy P, Forrest E (2016). Factors associated with spontaneous clearance of chronic hepatitis C virus infection. J Hepatol.

[34] Cuadrado A, Cobo C, Mateo M (2021). Telemedicine efficiently improves access to hepatitis C management to achieve HCV elimination in the penitentiary setting. Int J Drug Policy.

[35] Eckhardt B, Mateu-Gelabert P, Aponte-Melendez Y (2022). Accessible hepatitis C care for people who inject drugs: a randomized clinical trial. JAMA Intern Med.

[36] Høj SB, Jacka B, Minoyan N, Artenie AA, Bruneau J (2019). Conceptualising access in the direct-acting antiviral era: an integrated framework to inform research and practice in HCV care for people who inject drugs. Int J Drug Policy.

[37] Levander XA, Vega TA, Seaman A, Korthuis PT, Englander H (2021). Utilising an access to care integrated framework to explore the perceptions of hepatitis C treatment of hospital-based interventions among people who use drugs. Int J Drug Policy.

[38] Crawford S, Bath N (2013). Peer support models for people with a history of injecting drug use undertaking assessment and treatment for hepatitis C virus infection. Clin Infect Dis.

[39] Stagg HR, Surey J, Francis M (2019). Improving engagement with healthcare in hepatitis C: a randomised controlled trial of a peer support intervention. BMC Med.

[40] Zhao S, Liao X, Fan R (2022). An individualized cirrhosis screening strategy might be more cost-effective in the general population. J Hepatol.

[41] Talal AH, McLeod A, Andrews P (2019). Patient reaction to telemedicine for clinical management of hepatitis C virus integrated into an opioid treatment program. Telemed J E-Health.

[42] Grebely J, Hajarizadeh B, Lazarus JV, Bruneau J, Treloar C (2019). Elimination of hepatitis C virus infection among people who use drugs: ensuring equitable access to prevention, treatment, and care for all. Int J Drug Policy.

[43] Syed TA, Bashir MH, Farooqui SM (2019). Treatment outcomes of hepatitis C-infected patients in specialty clinic vs. primary care physician clinic: a comparative analysis. Gastroenterol Res Pract.

[44] Wade AJ, Doyle JS, Gane E (2020). Outcomes of treatment for hepatitis C in primary care, compared to hospital-based care: a randomized, controlled trial in people who inject drugs. Clin Infect Dis.

[45] Gunn J, Higgs P (2019). Pharmacy-led hepatitis C treatment pathways to help ensure elimination. Lancet Gastroenterol Hepatol.

[46] Kattakuzhy S, Gross C, Emmanuel B (2017). Expansion of treatment for hepatitis C Virus infection by task shifting to community-based nonspecialist providers: a nonrandomized clinical trial. Ann Intern Med.

[47] Papaluca T, McDonald L, Craigie A (2019). Outcomes of treatment for hepatitis C in prisoners using a nurse-led, statewide model of care. J Hepatol.

[48] Radley A, de Bruin M, Inglis SK (2020). Clinical effectiveness of pharmacist-led versus conventionally delivered antiviral treatment for hepatitis C virus in patients receiving opioid substitution therapy: a pragmatic, cluster-randomised trial. Lancet Gastroenterol Hepatol.

