# Supplementary material for: A randomized controlled trial for a peer-facilitated telemedicine hepatitis c treatment intervention for people who use drugs in rural communities: study protocol for the “peer tele-HCV” study
Source: Addict Sci Clin Pract. 2023 May 27;18:35. doi: 10.1186/s13722-023-00384-z (PMC10221743; doi:10.1186/s13722-023-00384-z)
Supplement: Supplementary file 2 — Additional file 2: Supplementary Material. [file 13722_2023_384_MOESM2_ESM.docx]

**Additional file 2**

**Consent Forms:**

CLINICAL RESEARCH CONSENT AND AUTHORIZATION SUMMARY OF KEY INFORMATION ABOUT THIS STUDY

**TITLE**: Oregon HIV/Hepatitis and Opioid Prevention and Engagement (OR-HOPE) Study: Tele-HCV Treatment Trial

**PRINCIPAL INVESTIGATOR**: Todd Korthuis, MD, MPH *(503) 494-8044*

You are being asked to join a research study. This consent form contains important information to help you decide if you want to join the study or not.

# PURPOSE:

The purpose of the study is to learn more about the use of telemedicine with help from a peer to treat hepatitis C for people who use drugs. Telemedicine is a way for healthcare providers to meet with patients remotely, over the phone or a computer. We are hoping to find out better ways to treat people living with both hepatitis C and a substance use disorder in the community setting.

# DURATION:

The purpose of the first portion of the study is to test your blood to see if you have Hepatitis

C. If you agree to the rapid blood test, and your blood comes back positive for Hepatitis C, then you may be eligible to participate in the full study. Your participation in the full study will consist of 5 visits over one year. Visits will last up to 30 minutes or however long you choose to meet with your doctor. We may ask to follow your health through the use of telemedicine appointments and surveys for up to one year.

# PROCEDURES:

If you decide to take part in the full study, you will be randomly assigned to a treatment arm for one of two treatments. Being randomly assigned is like flipping a coin; both the study staff and the participant cannot predict the treatment you will be assigned to, in the same way the result of a flipped coin is random and unpredictable. You participation in the randomly assigned treatment will last up to one year. You will be asked to have a number of tests and procedures. This includes:

- Rapid hepatitis C testing
- Telehealth appointments with a doctor to discuss hepatitis C treatment every four weeks

1

Version 1.0

- Surveys asking about symptoms, substance use and harm reduction every four weeks
- Two blood draws; one at the beginning of treatment and one at the end
- Genetic testing if the doctor decides it is necessary to provide the best treatment

# RISKS:

- Risk of underlying conditions: Other health issues that can have complications due to substance use treatment or hepatitis C treatment
- Risk to confidentiality
- Blood draw may cause pain or bruising at the site

**BENEFITS:** You may benefit from participation in the study by being diagnosed with HCV. You may also benefit by gaining access to treatment and cure for your diagnosis. The treatment may not be helpful for everyone.

**ALTERNATIVES:** You may choose not to participate in this study.

This is a voluntary research study. You do not have to join the study. Even if you decide to join now, you can change your mind later. Please ask the Investigator if you have any questions about the study or about this consent form.

# END OF CONSENT SUMMARY

2

# Clinical Research Consent and Authorization Form

**TITLE**: *Oregon HIV/Hepatitis and Opioid Prevention and Engagement (OR-HOPE) Study: Tele- HCV Treatment Trial*

**PRINCIPAL INVESTIGATOR**: P. Todd Korthuis (503) 494-8044

*.*

**WHO IS PAYING FOR THE STUDY?**: National Institutes of Health

# WHY IS THIS STUDY BEING DONE?:

You have been invited to be in this research study because you may have Hepatitis C and may be eligible to be treated through telemedicine hepatitis C treatment. The use of telemedicine may help provide access to those who cannot attend treatment in a doctor’s office, and therefore may be more helpful for you.

The purpose of the first portion of the study is to test your blood to see if you have Hepatitis C. If you agree to the rapid blood test, and your blood comes back positive for Hepatitis C, then you may be eligible to participate in the full study.

The full study requires 5 visits to the clinic and will take 12 weeks to complete, with a one year follow-up blood draw.

Up to 200 participants will be enrolled in this study with a group of 35 participants who will be randomly selected to complete an interview.

# WHAT EXAMS, TESTS AND PROCEDURES ARE INVOLVED IN THIS STUDY?

1. Prescreening: The prescreening portion of the study includes a rapid HCV test, which involves a finger prick. The results of the test will be available right away. You will have the option of completing the finger prick test on yourself. If you choose to do this, you will be given an instruction sheet. The research staff member will be with you to answer questions. You do not have to do the finger prick yourself. If you prefer, a research staff member will do the finger prick.

If your rapid HCV test comes back negative, you will be eligible for retesting in 6 months. If your rapid test is positive, and you want to participate in the full study, we will ask you a few additional questions to confirm that you are eligible. These questions are part of the study Inclusion/Exclusion list, and will take about five minutes to complete.

We will also ask you to fill out a form called a Locator Form. This will help us to find a way to contact you with the final results of your blood test.

1

Version 1.0

1. Surveys: You will be asked to complete a total of three separate surveys. If you enroll in the study before a particular date, you will also be asked to complete an additional survey. If you agree to complete the survey, you will be provided with more information at a later date. You will also be compensated. Questions asked in these surveys include: symptoms regarding your diagnosis, treatment satisfaction, substance use, and harm reduction engagement. You will complete this survey at your first appointment, four weeks after starting treatment, at the end of treatment, and 12 weeks after treatment is complete. We expect these surveys will last between 15-20 minutes.
   1. Optional Interview: We may also invite you to an in-person or telephone interview at any of these time points. This portion of the study is optional and you do not have to participant in the interview in order to participate in the study. If you do decide to participate in the interview, you will be given a separate information sheet consent form and will have an opportunity to ask questions about the interview. Interviews will last up to approximately 45 minutes. You will be asked questions about your experience using telehealth and HCV treatment.
2. Optional Future Contact: Researchers would like to be able to contact you about participating in possible future research studies. You do not have to consent to be contacted in the future. If you do not want to be contacted in the future, you can still participate in this study.
3. Lab Tests: A blood draw will be required to confirm your diagnosis. These results will be shared with you as it is a part of your normal medical care. About three tablespoons of blood will be drawn for this lab test. If not all the blood is drawn on the first blood draw, you may need to return to the lab for a second blood draw.
4. Treatment: If you enter the study and meet eligibility for treatment of your hepatitis C, your doctor will create a treatment plan with you.

This is a randomized study. Neither you nor the investigator can choose whether you will receive care through telemedicine or treatment as usual.

Your medical record will be reviewed by study staff in order to collect information relevant to the study, including the number of interactions with treatment staff.

# WILL I RECEIVE RESULTS FROM THE TESTING IN THIS STUDY?

We will give you the results of your rapid test and blood draw. The results will be placed in your medical record.

# WHAT RISKS CAN I EXPECT FROM TAKING PART IN THIS STUDY?:

**Rapid Test Risks:** During the rapid test, you may feel some pain in your finger.

**Risk of Underlying Conditions:** Depending on any underlying medical conditions you may have, you may experience complications of hepatitis C treatment.

**Risks to Confidentiality**: Although we will take steps to protect your personal information, there is a small risk of loss of confidentiality.

**Phlebotomy Risks:** We will draw blood from your arm. You may feel some pain when your blood is drawn. There is a small chance the needle will cause bleeding, a bruise, an infection, or fainting.

**Telemedicine Risks:** You may become uncomfortable while talking to a provider about your health issues.

**If you are diagnosed with Hepatitis C:** As a person living with hepatitis C, you can give hepatitis C to other people through any act that causes your blood to come into contact with theirs. This could include sharing any drug injection equipment, including needles, syringes, cotton/filters, rinses, or cookers. Anal receptive sex is another recognized way of sharing hepatitis C. You can protect yourself and others by always using new syringes, never sharing injection equipment with others, and wearing condoms during sex.

**Survey Risk:** Some of these questions may seem very personal or embarrassing. They may upset you. You may refuse to answer any of the questions that you do not wish to answer. If the questions make you very upset, we will help you to find a counselor.

# WHAT ARE THE BENEFITS OF TAKING PART IN THIS STUDY?

You may or may not benefit from being in this study. However, by serving as a subject, you may help us learn how to benefit patients in the future. You may benefit by being diagnosed with HCV.

# WHAT ARE MY CHOICES IF I DECIDE NOT TO TAKE PART IN THIS STUDY?

You may choose not to be in this study.

# WHO WILL SEE MY PERSONAL INFORMATION?

We will take steps to keep your personal information confidential, but we cannot guarantee total privacy. However, we will do our best to keep your information confidential by keeping it coded on an encrypted computer. Locator forms will be kept in locked cabinets that only the researchers have access to. We may request your social security number in order to process any payments for participation.

We will create and collect health information about you as described in the **WHY IS THIS STUDY BEING DONE?** and **the WHAT EXAMS, TESTS AND PROCEDURES ARE**

**INVOLVED IN THIS STUDY?** sections of this form. Health information is private and is protected under federal law and Oregon law. By agreeing to be in this study, you are giving permission (also called authorization) for us to use and disclose your health information as described in this form.

The investigators, study staff, and others at OHSU may use the information we collect and create about you in order to conduct and oversee this research study and store in a repository for optional future research.

We may have to release this information to others for example, if the study is audited. However, we would try to do so without information that could identify you. We may release this information to others outside of OHSU who are involved in conducting or overseeing research, including

- The funder of this study, National Institutes of Health and the funder’s representatives
- The Office for Human Research Protections, a federal agency that oversees research involving humans
- Comagine Health
- The Oregon Health Authority
- HIV Alliance
- Bay Area First Step
- Oregon Washington health Network
- Peace Health
- Mercy Medical Center
- Asante
- Interpath
- Bay Area Hospital
- Curry Health Network
- University of Washington

Those listed above may also be permitted to review and copy your records, including your medical records. If your information goes outside of OHSU, it might not be protected under federal law from being used or further shared. We would like your permission to keep your contact information and blood sample indefinitely.

We will not release information about you to others not listed above, unless required or permitted by law. We will not use your name or your identity for publication or publicity purposes, unless we have your special permission.

Under Oregon law, suspected child or elder abuse must be reported to appropriate authorities.

When we send specimens or information outside of OHSU, they may no longer be protected under federal or Oregon law. In this case, your specimens or information could be used and re- released without your permission.

Blood draw samples from this study may be shared with other investigators for future research studies. All identifying information about you will be removed from the samples before they are released to any other investigators. Results from the blood test will be sent to HIV Alliance and Molecular Testing Laboratories. Your name will not be associated with the blood samples.

Some of the information collected and created in this study may be placed in your OHSU medical record. While the research is in progress, you may or may not have access to this information. After the study is complete, you will be able to access any study information that was added to your OHSU medical record. If you have questions about what study information you will be able to access, and when, ask the investigator.

# WILL MY SENSITIVE INFORMATION RECEIVE ANY ADDITIONAL PROTECTIONS?

The law provides additional confidentiality protection for certain types of information, including drug and alcohol diagnosis, treatment, or referral information.

We will collect drug use and treatment information about you from the study surveys.

## WILL ANY OF MY INFORMATION OR SAMPLES FROM THIS STUDY BE USED FOR ANY COMMERCIAL PROFIT?

Information obtained from you in this research may be used for commercial purposes, such as making a discovery that could, in the future, be patented or licensed to a company, which could result in a possible financial benefit to that company, OHSU, and its researchers. There are no plans to pay you if this happens. You will not have any property rights or ownership or financial interest in or arising from products or data that may result from your participation in this study. Further, you will have no responsibility or liability for any use that may be made of your samples or information.

# WHAT ARE THE COSTS OF TAKING PART IN THIS STUDY?

Some of the services items in this study are part of the regular treatment for your condition. These would be performed or used even if you were not in the study. The cost for these services or items will be billed to your insurance. You will be responsible for any costs your insurance does not cover. If you have any questions about these costs, of what out of pocket expenses you may be responsible for, contact your insurance company. If you are uninsured, you will be responsible for these costs.

You will not be billed for the costs of any services or procedures that are required by the study but are not considered part of your regular treatment.

You will receive cash incentives for study activities you complete. The total amount that you could receive if you complete all study activities is $175.

**Compensation Schedule**

| Activity | Cash Incentive Amount |
| --- | --- |
| Prescreen/ Rapid Test/ Full Consent, Blood  draw | $20 |
| Screening blood draw | $20 |
| *Second blood draw if necessary* | $20 |
| Return for Results and Baseline Survey | $15 |
| 4 - Week survey | $20 |
| End of treatment survey | $30 |
| *12-weeks after end of treatment survey | $20 |
| *12-weeks after end of treatment blood draw | $40 |
| 36 weeks after end of treatment survey, reinfection labs (if qualified based on date of  enrollment) | $30 |
| Total | $215 |

# WHAT HAPPENS IF I AM INJURED BECAUSE I TOOK PART IN THIS STUDY?:

If you believe you have been injured or harmed as a result of participating in this research and require treatment, contact Todd Korthuis; 503-494-8044.

If you are injured or harmed by the study procedures, you will be treated. OHSU and the National Institutes of Health do not offer any financial compensation or payment for the cost of treatment if you are injured or harmed as a result of participating in this research. Therefore, any medical treatment you need may be billed to you or your insurance. However, you are not prevented from seeking to collect compensation for injury related to negligence on the part of those involved in the research. Oregon law (Oregon Tort Claims Act (ORS 30.260 through 30.300)) may limit the dollar amount that you may recover from OHSU or its caregivers and researchers for a claim relating to care or research at OHSU, and the time you have to bring a claim.

If you have questions on this subject, please call the OHSU Research Integrity Office at (503) 494-7887.

This federally funded study also does not have the ability to provide compensation for research- related injury. If you are injured or become ill from taking part in this study, it is important to tell your study doctor. Emergency treatment may be available but you or your insurance company will be charged for this treatment.

# WHERE CAN I GET MORE INFORMATION?

If you have any questions, concerns, or complaints regarding this study now or in the future, contact Todd Korthuis; (503) 494-8044**.**

This research has been approved and is overseen by an Institutional Review Board (“IRB”), a committee that protects the rights and welfare of research participants. You may talk to the IRB at (503) 494-7887 or [irb@ohsu.edu](mailto:irb@ohsu.edu) if:

- Your questions, concerns, or complaints are not being answered by the research team.
- You want to talk to someone besides the research team.
- You have questions about your rights as a research participant.
- You want to get more information or provide input about this research.

You may also submit a report to the OHSU Integrity Hotline online at <https://secure.ethicspoint.com/domain/media/en/gui/18915/index.html> or by calling toll-free (877) 733-8313 (anonymous and available 24 hours a day, 7 days a week).

# DO I HAVE TO TAKE PART IN THIS STUDY?

Your participation in this study is voluntary. You do not have to join this or any research study. You do not have to allow the use and disclosure of your health information in the study, but if you do not, you cannot be in the study. Some parts of the study are optional such as the interview and holding your blood draw data for future research. You can choose not to participate in some or all of the optional parts but still participate in the rest of the study.

# IF I DECIDE TO TAKE PART IN THIS STUDY, CAN I STOP LATER?

If you do join the study and later change your mind, you have the right to quit at any time. This includes the right to withdraw your authorization to use and disclose your health information.

You can choose to withdraw from some or all of the optional parts of this study without withdrawing from the whole study. If you choose not to join any or all parts of this study, or if you withdraw early from any or all parts of the study, there will be no penalty or loss of benefits to which you are otherwise entitled, including being able to receive health care services or insurance coverage for services. Talk to the investigator if you want to withdraw from the study or change which parts of the study you are participating in.

If you no longer want your health information to be used and disclosed as described in this form, you must send a written request or email stating that you are revoking your authorization to:

Todd Korthuis

(503) 494-4088

3181 S.W. Sam Jackson Park Road Portland, Oregon 97239

Your request will be effective as of the date we receive it. However, health information collected before your request is received may continue to be used and disclosed to the extent that we have already acted based on your authorization.

The blood draw we will collect from you will not be stored with your name or any other identifier. Therefore, there will not be a way for us to identify and destroy your materials if you decide in the future that you do not wish to participate in this research.

You may be removed from the study if the principle investigator or funder stops the study, or if you do not follow study instructions.

We will give you any new information during the course of this research study that might change the way you feel about being in the study.

# SIGNATURES:

***PARTICIPANT OPTIONS***

The optional portions of this study are described in detail throughout this consent form and listed here as a summary. Please read the options and place your initials next to your choices You can still participate in the main part of the study even if you choose not to participate in the optional parts.

I give my consent to be potentially selected for an interview.

I agree to be contacted about future study participation

Your signature below indicates that you have read this entire form and that you agree to be in this study.

We will give you a copy of this signed form.

Participant Printed Name Participant Signature Date

Person Obtaining Consent Printed Name Person Obtaining Consent Signature Date

**Table S1: Lab Determination Form**

| **Hepatitis C Lab Form Determination Work Sheet** | | |
| --- | --- | --- |
| Ask the client the following **yes** or **no** questions | **Yes**  circle the number below if client answers ‘yes’ | **No**  circle the number below if client answers ‘no’ |
| **Have you had any Hepatitis C treatment before?**  (completed treatment and  re-infected, did not complete treatment) | 1 | 5 |
| **Are you someone with a uterus?** | 2 | 4 |
| **Are you over the age of 50?** | 3 | 6 |
| Add the **TOTAL** of the **CIRCLED** numbers together | | |
| **Enter the total here:** | |  |

| **Total Number** | **Lab Order Form** |
| --- | --- |
| **Total= 13** (treatment naive and women under 50) | **[LabName]_[ProviderName]_A** |
| **Total =15, 12, 10** (treatment naive and no pregnancy) | **[LabName]_[ProviderName]_B** |
| **Total = 9** (treatment experienced and women under 50) | **[LabName]_[ProviderName]_C** |
| **Total= 6, 8, 11** (treatment experienced and no pregnancy) | **[LabName]_[ProviderName]_D** |

**Use the key below to determine what standing lab form your client will need**

**Table S2: Physician Screening Confirmation for Child Pugh ≥ 6 or Platelets < 160,000**

| **History**   1. Current swelling in legs? If so, explain. (yes / yes but inconsistent with decompensated cirrhosis / no): *** 2. Current swelling in abdomen? If so, explain. (yes / yes but inconsistent with decompensated cirrhosis / no): *** 3. Confusion? If so, explain. (yes / yes but inconsistent with decompensated cirrhosis / no): *** 4. Hx suggestive of asterixis (yes/no): ***   Meets inclusion criteria (yes/no): ***  Meets exclusion criteria (yes/no): ***  **Yes answer for 1-4 considered consistent with current decompensated cirrhosis by the study clinician may meet exclusion criteria, unless findings better explained by study clinician discretion.* |
| --- |
